# Method overtness, forensic autopsy, and the evidentiary suicide note: A multilevel National Violent Death Reporting System analysis

**DOI:** 10.1371/journal.pone.0197805

**Published:** 2018-05-22

**Authors:** Ian R. H. Rockett, Eric D. Caine, Steven Stack, Hilary S. Connery, Kurt B. Nolte, Christa L. Lilly, Ted R. Miller, Lewis S. Nelson, Sandra L. Putnam, Paul S. Nestadt, Haomiao Jia

**Affiliations:** 1 Department of Epidemiology, West Virginia University, Morgantown, West Virginia, United States of America; 2 Injury Control Research Center, West Virginia University, Morgantown, West Virginia, United States of America; 3 Department of Psychiatry, University of Rochester Medical Center, Rochester, New York, United States of America; 4 Injury Control Research Center for Suicide Prevention, University of Rochester Medical Center, Rochester, New York, United States of America; 5 Department of Criminal Justice, Wayne State University, Detroit, Michigan, United States of America; 6 Department of Psychiatry and Behavioral Neuroscience, Wayne State University, Detroit, Michigan, United States of America; 7 Division of Alcohol and Drug Abuse, McLean Hospital, Boston, Massachusetts, United States of America; 8 Department of Psychiatry, Harvard Medical School, Boston, Massachusetts, United States of America; 9 Office of the Medical Investigator, University of New Mexico School of Medicine, Albuquerque, New Mexico, United States of America; 10 Department of Biostatistics, West Virginia University, Morgantown, West Virginia, United States of America; 11 Pacific Institute for Research and Evaluation, Calverton, Maryland, United States of America; 12 Curtin University School of Public Health, Perth, Australia; 13 Department of Emergency Medicine, Rutgers New Jersey Medical School, Newark, New Jersey, United States of America; 14 Department of Mental Health, Johns Hopkins Bloomberg School of Public Health, Baltimore, Maryland, United States of America; 15 Department of Psychiatry and Behavioral Sciences, Johns Hopkins University School of Medicine, Baltimore, Maryland, United States of America; 16 School of Nursing, Columbia University, New York, New York, United States of America; 17 Department of Biostatistics, Mailman School of Public Health, Columbia University, New York, New York, United States of America; Max-Planck-Institut fur auslandisches und internationales Strafrecht, GERMANY

## Abstract

**Objective:**

Higher prevalence of suicide notes could signify more conservatism in accounting and greater proneness to undercounting of suicide by method. We tested two hypotheses: (1) an evidentiary suicide note is more likely to accompany suicides by drug-intoxication and by other poisoning, as less violent and less forensically overt methods, than suicides by firearm and hanging/suffocation; and (2) performance of a forensic autopsy attenuates any observed association between overtness of method and the reported presence of a note.

**Methods:**

This multilevel (individual/county), multivariable analysis employed a generalized linear mixed model (GLMM). Representing the 17 states participating in the United States National Violent Death Reporting System throughout 2011–2013, the study population comprised registered suicides, aged 15 years and older. Decedents totaled 32,151. The outcome measure was relative odds of an authenticated suicide note.

**Results:**

An authenticated suicide note was documented in 31% of the suicide cases. Inspection of the full multivariable model showed a suicide note was more likely to manifest among drug intoxication (adjusted odds ratio [OR], 1.70; 95% CI, 1.56, 1.85) and other poisoning suicides (OR, 2.12; 1.85, 2.42) than firearm suicides, the referent. Respective excesses were larger when there was no autopsy or autopsy status was unknown (OR, 1.86; 95% CI, 1.61, 2.14) and (OR, 2.25; 95% CI, 1.86, 2.72) relative to the comparisons with a forensic autopsy (OR, 1.62, 95% CI, 1.45, 1.82 and OR, 2.01; 95% CI, 1.66, 2.43). Hanging/suffocation suicides did not differ from the firearm referent given an autopsy.

**Conclusions:**

Suicide requires substantial affirmative evidence to establish manner of death, and affirmation of drug intoxication suicides appears to demand an especially high burden of proof. Findings and their implications argue for more stringent investigative standards, better training, and more resources to support comprehensive and accurate case ascertainment, as the foundation for developing evidence-based suicide prevention initiatives.

## Introduction

Emerging as the leading cause of injury mortality in the United States (US) by 2009 [1], suicide is not a default manner-of-death determination for medical examiners and coroners [2–4]. A socially condemned and stigmatized phenomenon, suicides are undercounted [5], with false negativity posing a far greater threat than false positivity to valid certification [6,7]. National and international research indicates that undercounting is nonrandom across suicide methods [8,9]. The three leading methods of suicide in the US are firearm, hanging, and poisoning. Collectively, these methods accounted for 92% of total suicides in 2015 [10]. Suicides by poisoning are likely far more challenging for medical examiners and coroners to ascertain than those by shooting and hanging [4,11–14]. Gross discrepancies among these major methods in accounting for suicide would inhibit, or even preclude, accurate risk group delineation and risk factor identification, and hence impede the design, targeting, and implementation of effective clinical and population interventions.

Availability of a suicide note or an equivalent—whether written, typed, digital, or audio [15]—can serve as a pivotal piece of external forensic and psychological evidence for determining suicide as the manner of death [16]. Some countries even require a suicide note to record a death as suicide [17], as was true of some US coroners in the 1970s [18]. Thus, lack of an evidentiary note may induce suicide misclassification in the US [19]. Echoing its pivotal nature, a US study found that a suicide manner of death was associated with a far higher prevalence of notes than was undetermined intent, 32% versus 1.5% [20]. Two English studies also revealed large prevalence gaps [21,22]. Undetermined is the manner of death category most susceptible to obscuring suicides [21,23–25], in relative terms, with poisoning its predominant underlying cause-of-death sub-category in the US [14,26].

Two earlier American studies found an excess of note-leaving among suicides by poisoning relative to other methods [27,28], whereas a later one found an excess among hanging suicides [29]. A subsequent individual-level, multivariable analysis of National Violent Death Reporting System (NVDRS) data, for the period 2003–2006, showed excess note-leaving among suicides by poisoning, firearm, and hanging versus all other methods except jumping from a height and drowning, two other less forensically overt methods [30]. That study also showed note leaving was nonrandom across demographic characteristics such as age, sex, race/ethnicity, marital status, and urban residence. Finally, an Austrian study found no difference in the prevalence of note-leaving across methods of suicide or age, sex, family status, psychiatric care or motive [31]. Unlike the US, Austria is among rare countries whose suicide certification appears very accurate [32,33].

In this multilevel, multivariable study of NVDRS data for 2011–2013, we posited that a higher proportion of suicide notes in manner of death determinations involving fatal drug introxication, as compared to those involving a firearm or hanging, reflected their use as key evidence in the differential process of establishing suicide as the manner of death. This could signify stricter (i.e., more conservative) evidentiary standards when making these determinations, leading in turn to a greater potential for undercounting suicides.

We addressed two questions in our examination of the differential determination of suicide, with a view to informing and improving surveillance, etiologic understanding, and prevention of suicide and related injury mortality. Was an evidentiary note more common among suicide cases involving drug intoxication and other poisoning, less self-evident causes and manner of death, as compared to the violent methods of firearm and hanging, where apparent motivation is more overt? Did the performance of a forensic autopsy serve to mediate the association between the reporting of a note and the overtness of the suicide method? Although a forensic autopsy is not a personal window into decedent intent [34], together with toxicology it generates evidence that helps medical examiners and coroners identify injury mechanisms (i.e., causes of death) and decedent intent in suspicious or uncertain cases [35–37].

Salient to our study, forensic autopsy and toxicological testing rates vary greatly across states [38,39]. Since suicides are local events, and often socially stigmatized [40,41], we adjusted our analyses for both county-level and individual-level factors. They comprised characteristics of the medicolegal death investigations and the state-county investigation systems, decedent and areal demographics, and documented mental health antecedents and other death circumstances.

## Data and methods

### Individual-level data source and variables

The source for our individual-level variables was the Restricted Access Database from the NVDRS, which is administered by the Centers for Disease Control, and Prevention (CDC). This state, territory, and incident-based surveillance system employs public health informatics for making data linkages, primarily among death certificates, law enforcement records, and medical examiner and coroner records. Also variably incorporating such optional supplements as laboratory reports and hospital records, the NVDRS provides de-identified information about suicide and other violent deaths, including their geographic location, circumstances, and personal sociodemographics, in addition to investigation specifics [42–44]. Disaggregatable to county of death, the data analyzed in this study represented the 17 states that participated in the NVDRS throughout the 2011–2013 observation period. They were Alaska, Colorado, Georgia, Kentucky, Maryland, Massachusetts, New Jersey, New Mexico, North Carolina, Ohio, Oklahoma, Oregon, Rhode Island, South Carolina, Utah, Virginia, and Wisconsin. Enhancing study generalizability, these states mirrored the nation in their age and sex composition, manner-of-death distribution, and crude and age-adjusted all-cause, suicide, and undetermined intent death rates [20]. They overrepresented non-Hispanic Blacks and Whites and underrepresented Hispanics.

Our study population comprised registered suicides (ICD-10: U03, X60-X84, Y87.0), whose method and state and county of death were specified, and was further confined to decedents aged 15 years and older, since fewer than 1% of known suicides were younger. The number of decedents in our multivariable analyses totaled 32,151. As further background, our first table provides comparative data on suicide methods for the 17 NVDRS states and the US. These comparisons were based on separate counts for the selected demographic variables, not study population and corresponding national population counts. Similarity between the states and the nation would fortify the generalizability of the results. The data source was CDC WISQARS™ (Web-based Injury Statistics Query and Reporting System) [10].

The outcome variable in this study was suicide note (yes versus no or unknown). Our predictor distinguished suicide methods as follows: drug intoxication, other poisoning, jumping and drowning (combined owing to small sample size and similar corroborative challenges), hanging/suffocation, firearm (referent), and all other methods specified in the NVDRS suicide cases. Additional individual-level covariates were prior suicide attempt; primary mental diagnosis; current mental health treatment; blood alcohol concentration; number of other specified drug positives; physical health problem; number of intimate partner or legal or job or financial or school problems; crisis in past two weeks; emergency medical services at scene; region of death; age; sex; race/ethnicity; marital status; education; and military veteran status. The mediator was autopsy status (yes versus no or unknown).

### County-level data sources and covariates

Linked to the individual-level data in this study, our county-level death investigation system covariates were mode of selection of the chief medical examiner or coroner for a given system (elected versus appointed) and accreditation status and type (accredited coroner, accredited medical examiner, unaccredited coroner, unaccredited medical examiner). Selection mode was identified through a CDC website [45], and accreditation status and system type through the respective websites of the relevant accrediting agencies, the National Association of Medical Examiners (NAME) [46] and the International Association of Coroners and Medical Examiners (IACME) [47]. We resolved outstanding questions by email or telephone communication with state, district, or county offices. County-level demographic covariates were urbanicity (5 categories representing the 12 ordinal categories of the 2013 Urban Influence Codes: large metropolitan/small metropolitan/metropolitan adjacent/micropolitan or adjacent/rural) and percent population below poverty as the measure of the local poverty burden. These two covariates served as proxies for external forces that potentially could inhibit or support medicolegal death investigations [48]. Their source was the County Area Health Resource File for 2014–2015 [49].

### Hypotheses and statistical approach

We used a generalized linear mixed model (GLMM) to test the following two hypotheses: (1) an evidentiary suicide note is more likely to accompany suicides by drug-intoxication and by other poisoning than suicides by firearm and hanging/suffocation; and (2) performance of a forensic autopsy attenuates the observed association between overtness of method and the reported presence of a note. In testing the first hypothesis, we progressively applied four models, beginning with a univariable analysis, confined to suicide method, followed by multivariable analyses that cumulatively incorporated medicolegal system and investigation characteristics, mental health antecedents and precipitating circumstances, and decedent and areal demographics. Providing for a fifth multivariable model, we then tested our second hypothesis. The GLMM is a two-level model that is logistic at the individual level and linear at the county level. We also included a state-level random effect to incorporate the data structure of counties nested in a state and individuals nested in a county. The statistical software was SAS (Copyright(c) 2002–2010, SAS Institute Inc., Cary, NC).

## Results

Table 1 describes the frequency of suicide by method across age, sex, and race/ethnicity for the NVDRS states and nationally during the observation period. Firearm, hanging, and drug intoxication were the leading methods of suicide. Generally, the states and nation showed very similar percentage distributions across age and sex, except at ages 14 years and under. The small numbers of the latter induced data instability; this age group was eliminated from our study population.

**Table 1:** Percent suicide by method and selected demographic characteristics: 17 National Violent Death Reporting System States and the United States, 2011–2013^a^.

Characteristic/ Method	Firearm		Hanging/ Suffocation		Drug Intoxication		Other Poisoning		Jumping/ Drowning		Other Methods^b^		n	N
*Age (years)*	NVDRS	US	NVDRS	US	NVDRS	US	NVDRS	US	NVDRS	US	NVDRS	US	NVDRS	US
All ages	52.3	51.0	24.4	24.8	13.2	13.3	3.1	3.1	2.6	3.2	4.2	4.6	37,909	121,267
0–14	18.5	33.7	53.2	62.2	0	2.4	0	0	0	0.8	28.3	0.8	314	993
15–34	47.0	45.1	35.1	35.8	8.6	8.5	2.0	2.0	2.3	3.8	5.0	4.7	10,809	33,236
35–54	47.0	45.3	25.3	25.9	16.6	16.8	3.7	3.8	2.2	3.2	5.2	5.0	14,605	46,249
55–74	60.2	58.5	14.0	14.9	15.1	15.8	3.3	3.4	1.5	2.9	5.9	4.5	9,422	30,925
75+	76.9	75.4	7.6	9.6	5.6	7.1	0.4	2.5	0	2.8	9.5	2.6	2,741	9,844
***Sex***														
female	32.9	31.6	23.1	23.5	32.6	32.5	3.0	3.4	2.3	4.3	6.1	4.7	8,406	26,429
male	57.8	56.4	24.8	25.2	7.7	8.0	3.1	3.0	2.3	2.9	4.3	4.5	29,503	94,838
***Race/ethnicity***														
White non-Hispanic	53.9	53.6	22.6	22.2	13.8	14.1	3.2	3.2	2.4	2.7	4.1	4.2	32,443	101,011
Hispanic	36.4	35.7	41.5	41.5	8.0	9.9	0	1.7	1.0	4.4	13.1	6.7	1,627	8,125
Black non-Hispanic	49.2	47.8	26.9	28.4	7.3	9.2	1.9	2.3	0.9	3.6	13.8	8.7	2,419	6,951
Other	34.7	27.6	40.7	46.0	5.4	9.8	0	3.0	0.8	6.9	18.4	6.7	1,332	4,781

^a^ The data source was the Web-based Injury Statistics Query and Reporting System (WISQARS™), administered by the Centers for Disease Control and Prevention.

^b^ Comprises both specified and unspecified suicide methods.

Bivariable frequency data on suicide notes for the study population are presented in Table 2. An authenticated suicide note was documented in 31% of the suicide cases. Consistent with our principal hypothesis, an excess prevalence of notes manifested for suicides by drug intoxication and by other poisoning, at 42% and 48%, versus 29% and 31% for firearm and hanging/suffocation suicides, respectively. Pertinent to statistical adjustments in the multivariable analyses, note prevalence reflected some marked variability by selection mode of the chief medical examiner/coroner, blood alcohol concentration, region of death, and within the sociodemographic characteristics of sex, race/ethnicity, marital status, and education.

**Table 2:** Prevalence of suicide notes by decedent demographics, death circumstances, and investigation system characteristics, 17 National Violent Death Reporting System States, 2011–2013.

Demographics	%	n^a^	Circumstances	%	n^a^	Investigation	%	n^a^
***Age (years)***			***Past suicide attempt***			***Chief medical examiner/coroner***		
15–34	28.3	9,147	yes	34.9	5,858	elected	27.4	11,448
35–54	32.7	12,525	no/unknown	30.7	26,293	appointed	33.6	20,703
55–74	32.9	8,125	***Mental diagnosis***			***System accreditation/type***		
75+	28.3	2,354	depression/dysthymia	35.0	9,313	accredited medical examiner	34.4	8,479
**Total**	31.4	32,151	anxiety disorder	38.4	539	coroner	36.6	1,685
***Sex***			bipolar disorder	34.5	1,209	unaccredited medical examiner	33.2	12,269
male	29.6	25,100	other	23.2	764	coroner	33.2	9,718
female	37.8	7,051	none/unknown	29.7	20,326	***Method***		
***Race/ethnicity***			***Mental health treatment***			drug intoxication	42.2	4,154
White non-Hispanic	32.7	27,333	yes	34.2	9,850	other poisoning	48.2	1,014
Hispanic	26.5	1,149	no/unknown	30.2	22,301	firearm	28.7	16,609
Black non-Hispanic	19.6	1,691	***Physical health problem***			hanging	30.6	8,017
other	26.8	1,978	yes	37.5	6,010	jumping/drowning	29.1	824
***Marital status***			no/unknown	30.0	26,141	all other	25.6	1,533
single	30.2	10,891	***Personal problems***			***Blood alcohol concentration***		
married	29.9	11,344	0/unknown	28.2	17,565	0.00 g/dl	34.9	10,946
widowed/divorced/separated	34.5	9,655	1	34.3	10,134	0.10–0.79	37.7	1,921
unknown	31.8	261	2	37.8	3,422	no test/unknown	29.1	14,260
***Education (years)***			3+	37.2	1,030	> = 0.80	28.1	5,024
0–8	15.0	1,154	***Recent crisis***			***Other drug positive***		
9–12	25.1	1,704	yes	33.8	3,558	0	30.4	23,860
13+	33.1	25,216	no/unknown	31.1	28,593	1	33.7	4,914
unknown	28.4	4,077	***Emergency medical services***			2	35.5	2,209
***Military veteran***			present at scene	32.6	23,377	3+	35.8	1,168
yes	31.7	5,795	absent/unknown	28.4	8,774	***Autopsy***		
no/unknown	31.4	26,356				yes	30.7	16,409
***Region of death***						no/unknown	32.2	15,742
other	34.0	18,990						
South	27.7	13,161						

^a^ Total number of cases within each variable subcategory.

Partially affirming the principal hypothesis, the univariable analysis showed drug intoxication suicides and suicides by other poisoning were respectively 71% and 122% more likely than their referent, firearm suicides, to be associated with a note (Table 3). Neither hanging suicides, as consistent with our hypothesis, nor combined jumping and drowning suicides deviated from the referent. Findings remained robust as grouped death investigation variables (Model 2), precipitating circumstances (Model 3), and decedent and county-level demographics (Model 4) were cumulatively incorporated into the multivariable analyses. For drug intoxication suicides, percent increase in odds of a note relative to the firearm referent rose from 71% in the univariable analysis to 80% and 88%, respectively, in the multivariable analysis with application of Models 2 and 3, and diminution to 66% following addition of the demographics in Model 4. Corresponding changes were more modest for the other poisoning suicide category. However, under Models 1 and 4, percent increase in odds of a note relative to firearms was larger for other poisoning suicides than for drug intoxication suicides at 122% and 113%, respectively.

**Table 3:** Odds ratios and 95% confidence intervals for an evidentiary note by suicide method, cumulatively adjusted for investigation characteristics, death circumstances, and demographics, 17 National Violent Death Reporting System States, 2011–2013.

	Model 1	Model 2	Model 3	Model 4
Category/*Variable*^a^	OR (95% CI)	OR (95% CI)	OR (95% CI)	OR (95% CI)
***Suicide method***				
drug intoxication	1.71 (1.60, 1.84)^d^	1.80 (1.66, 1.95)^d^	1.88 (1.73, 2.04)^d^	1.66 (1.53, 1.81)^d^
other poisoning	2.22 (1.94, 2.52)^d^	2.21 (1.94, 2.52)^d^	2.25 (1.97, 2.57)^d^	2.13 (1.87, 2.44)^d^
jumping/drowning	0.93 (0.79, 1.08)	0.91 (0.78, 1.07)	1.00 (0.86, 1.18)	0.98 (0.83, 1.15)
hanging	1.04 (0.97, 1.10)	1.04 (0.97, 1.10)	1.05 (0.99, 1.12)	1.07 (1.00, 1.14)
other	0.81 (0.72, 0.92)^c^	0.81 (0.71, 0.91)^c^	0.86 (0.76, 0.97)^b^	0.83 (0.73, 0.94)^c^
firearm	1.00	1.00	1.00	1.00
**Investigation**				
***System accreditation/type***				
accredited medical examiner		1.46 (0.90, 2.36)	1.41 (0.90, 2.20)	1.37 (0.93, 2.02)
coroner		1.48 (1.16, 1.89)^c^	1.42 (1.13, 1.78)^c^	1.38 (1.12, 1.69)^c^
unaccredited medical examiner		1.34 (0.82, 2.20)	1.32 (0.83, 2.08)	1.22 (0.81, 1.83)
coroner		1.00	1.00	1.00
***Blood alcohol concentration***				
0.00 g dl		1.36 (1.26, 1.46)^d^	1.37 (1.27, 1.48)^d^	1.39 (1.29, 1.50)^d^
0.10–0.79		1.50 (1.33, 1.68)^d^	1.52 (1.36, 1.71)^d^	1.55 (1.38, 1.74)^d^
no test/unknown		1.20 (1.11, 1.30)^d^	1.22 (1.13, 1.33)^d^	1.23 (1.13, 1.33)^d^
≥ = 0.80		1.00	1.00	1.00
***Drug positives***				
0/no test or unknown		1.27 (1.11, 1.46)^c^	1.28 (1.12, 1.48)^c^	1.33 (1.15, 1.53)^d^
1		1.17 (1.01, 1.34)^b^	1.18 (1.02, 1.36)^b^	1.21 (1.05, 1.40)^c^
2		1.13 (0.97, 1.31)	1.12 (0.96, 1.30)	1.13 (0.97, 1.32)
3+		1.00	1.00	1.00
**Circumstances**				
***Mental diagnosis***				
depression/dysthymia			1.09 (1.01, 1.18)^b^	1.05 (0.97, 1.13)
anxiety disorder			1.21 (1.00, 1.46)^b^	1.17 (0.96, 1.41)
bipolar disorder			1.04 (0.91, 1.19)	0.99 (0.86, 1.14)
other			0.65 (0.55, 0.78)^c^	0.66 (0.55, 0.79)^d^
none/unknown			1.00	1.00
***Mental health treatment***				
yes			1.07 (0.99, 1.15)	1.09 (1.01, 1.18)^b^
no/unknown			1.00	1.00
***Physical health problem***				
yes			1.41 (1.32, 1.50)^d^	1.36 (1.27, 1.46)^d^
no/unknown			1.00	1.00
***Personal problems***				
1			1.34 (1.26, 1.42)^d^	1.39 (1.31, 1.48)^d^
2			1.51 (1.39, 1.64)^d^	1.57 (1.44, 1.71)^d^
3+			1.45 (1.26, 1.66)^d^	1.50 (1.31, 1.48)^d^
none/unknown			1.00	1.00
***Recent crisis***				
yes			1.10 (1.02, 1.19)^b^	1.08 (0.99, 1.17)
no/unknown			1.00	1.00
**Demographics**				
***Age (years)***				
35–54				1.14 (1.06, 1.22)^c^
55–74				1.15 (1.06, 1.25)^c^
75+				1.14 (1.00, 1.29)
15–34				1.00
***Sex***				
female				1.36 (1.27, 1.45)^d^
male				1.00
***Race/ethnicity***				
White non-Hispanic				1.94 (1.71, 2.21)^d^
Hispanic				1.49 (1.23, 1.81)^d^
other				1.56 (1.32, 1.84)^d^
Black non-Hispanic				1.00
***Marital status***				
single				1.21 (1.13, 1.30)^d^
widowed/divorced/separated				1.21 (1.14, 1.29)^d^
unknown				1.28 (0.97, 1.69)
married				1.00
***Education (years)***				
9–12				1.26 (1.02, 1.56)^b^
13+				1.73 (1.44, 2.07)^d^
unknown				1.28 (0.97, 1.69)
0–8				1.00
***Military veteran***				
yes				1.11 (1.03, 1.19)^c^
no				1.00
***Region of death***				
other				1.26 (1.00, 1.59)
South				1.00
***County urbanicity***				
metropolitan large				1.34 (1.06, 1.71)
metropolitan small				1.39 (1.11, 1.74)^c^
metropolitan adjacent				1.16 (0.93, 1.46)
micropolitan or adjacent				1.00 (0.73, 1.36)
rural				1.00
***County poverty (%)***				0.98 (0.97, 0.99)^d^

^a^ For economy, the table excludes three variables that were incorporated in the multivariable analysis, but showed no association with the outcome variable. They were chief medical examiner/coroner selection, prior suicide attempt, and emergency medical services at death scene.

^b^ p < 0.05

^c^ p < 0.01

^d^ p < 0.001

The stratified analysis supported our second hypothesis as performance of a forensic autopsy attenuated the association between suicide method and suicide note outcome (Table 4). Percent increase in odds of an evidentiary note for drug intoxication suicides, relative to firearm suicides, was 86% with no autopsy or unknown autopsy status versus 62% with autopsy. Corresponding figures for other poisoning suicides were 125% and 101%. With no autopsy or unknown autopsy status, hanging/suffocation suicides were less likely than firearm referents to be accompanied by a suicide note. Expanding Model 4 to include autopsy status showed increased percent odds of a note for drug intoxication suicides and other poisoning suicides of 70% and 112%, respectively, and a diminished effect for the residual group, all other suicide methods, compared to the firearm referent. Further inspection of the full model revealed that cases with no autopsy or with unknown autopsy status had 26% higher odds than autopsied cases of having a suicide note on record.

**Table 4:** Odds ratios and 95% confidence intervals for an evidentiary note by suicide method, stratified by autopsy status and unstratified, adjusted for investigation characteristics, death circumstances, and demographics, 17 National Violent Death Reporting System States, 2011–2013.

	Autopsy				Full Model
Category/*Variable*^a^	Yes		No/unknown		includes autopsy status
	OR (95%CI)	n	OR (95%CI)	n	OR (95%CI)
***Suicide method***					
drug intoxication	1.62 (1.45, 1.82) ^d^	2,866	1.86 (1.61, 2.14) ^d^	1,288	1.70 (1.56, 1.85) ^d^
other poisoning	2.01 (1.66, 2.43) ^d^	512	2.25 (1.86, 2.72) ^d^	502	2.12 (1.85, 2.42) ^d^
jumping/drowning	1.04 (0.85, 1.27)	527	0.96 (0.73, 1.25)	297	0.99 (0.85, 1.17)
hanging/suffocation	0.86 (0.73, 1.01)	3,365	0.80 (0.65, 0.97) ^b^	4,652	1.04 (0.97, 1.11)
other	1.00 (0.91, 1.11)	939	1.07 (0.98, 1.17)	594	0.83 (0.74, 0.95) ^c^
firearm	1.00	8,200	1.00	8,409	1.00
**Investigation**					
***System accreditation/type***					
accredited medical examiner	1.32 (0.91, 1.90)	5,836	1.80 (0.73, 4.44)	2,643	1.42 (0.96, 2.10)
accredited coroner	1.48 (1.20, 1.82) ^c^	1,385	1.28 (0.88, 1.88)	300	1.43 (1.17, 1.76) ^c^
unaccredited medical examiner	1.11 (0.74, 1.65)	3,931	1.79 (0.72, 4.42)	8,338	1.18 (0.79, 1.77)
unaccredited coroner	1.00	5,257	1.00	4,461	1.00
***Autopsy***					
no/unknown					1.26 (1.18, 1.34) ^d^
yes					1.00
***Blood alcohol concentration***					
0.00 mg/dl	1.45 (1.31, 1.61) ^d^	5,832	1.35 (1.20, 1.51) ^d^	5,114	1.39 (1.29, 1.51) ^d^
0.10–0.79	1.63 (1.41, 1.89) ^d^	1,236	1.50 (1.24, 1.80) ^d^	685	1.56 (1.39, 1.75) ^d^
no test/unknown	1.28 (1.14, 1.42) ^d^	6481	1.11 (0.98, 1.26)	7,779	1.20 (1.10, 1.30) ^d^
> = 0.80	1.00	2,860	1.00	2,164	1.00
***Other drug positive***					
0/no test or unknown	1.28 (1.08, 1.51) ^c^	10,657	1.24 (0.96, 1.62)	13,203	1.28 (1.11, 1.47) ^c^
1	1.22 (1.03, 1.45) ^b^	3,328	1.11 (0.85, 1.46)	1,586	1.19 (1.03, 1.38) ^b^
2	1.16 (0.96, 1.39)	1,555	1.04 (0.77, 1.39)	654	1.13 (0.96, 1.31)
3+	1.00	869	1.00	299	1.00
**Circumstances**					
***Mental diagnosis***					
depression/dysthymia	1.02 (0.92, 1.14)	4,646	1.05 (0.94, 1.18)	4,667	1.04 (0.96, 1.12)
anxiety disorder	1.12 (0.88, 1.43)	336	1.22 (0.90, 1.65)	203	1.17 (0.97, 1.41)
bipolar disorder	1.07 (0.89, 1.29)	709	0.89 (0.71, 1.10)	500	0.99 (0.86, 1.14)
other	0.68 (0.53, 0.88) ^c^	403	0.60 (0.46, 0.79) ^c^	361	0.65 (0.54, 0.78) ^d^
none/unknown	1.00	10,315	1.00	10,011	1.00
***Mental health treatment***					
yes	1.08 (0.96, 1.20)	5,095	1.11 (0.99, 1.24)	4,755	1.09 (1.01, 1.18) ^b^
no/unknown	1.00	1,314	1.00	10,987	1.00
***Physical health problem***					
yes	1.40 (1.27, 1.53) ^d^	3,022	1.31 (1.19, 1.45) ^d^	2,988	1.36 (1.27, 1.46) ^d^
no/unknown	1.00	13,387	1.00	12,754	1.00
***Personal problems***					
1	1.44 (1.33, 1.56) ^d^	5,338	1.36 (1.25, 1.48) ^d^	4,796	1.40 (1.32, 1.48) ^d^
2	1.72 (1.53, 1.94) ^d^	1,804	1.44 (1.27, 1.62) ^d^	1,618	1.58 (1.45, 1.72) ^d^
3+	1.60 (1.31, 1.94) ^d^	540	1.42 (1.16, 1.74) ^c^	490	1.51 (1.31, 1.74) ^d^
none/unknown	1.00	8,727	1.00	8,838	1.00
**Demographics**					
***Age (years)***					
35–54	1.21 (1.09, 1.33) ^c^	4,780	1.06 (0.96, 1.18)	4,367	1.13 (1.06, 1.22) ^c^
55–74	1.24 (1.10, 1.39) ^c^	6,540	1.06 (0.94, 1.20)	5,985	1.15 (1.05, 1.24) ^c^
75+	1.17 (0.98, 1.41)	4,097	1.07 (0.90, 1.27)	4,028	1.12 (0.99, 1.27)
15–34	1.00	992	1.00	1,362	1.00
***Sex***					
female	1.41 (1.29, 1.54) ^d^	4,035	1.33 (1.21, 1.46) ^d^	3,016	1.37 (1.28, 1.46) ^d^
male	1.00	12,374	1.00	12,726	1.00
***Race/ethnicity***					
White non-Hispanic	2.18 (1.85, 2.59) ^d^	13,557	1.55 (1.26, 1.90) ^d^	13,776	1.92 (1.68, 2.18) ^d^
Hispanic	1.67 (1.32, 2.13) ^d^	798	1.22 (0.89, 1.67)	351	1.48 (1.23, 1.79) ^d^
other	1.82 (1.46, 2.27) ^d^	943	1.22 (0.95, 1.56)	1,035	1.53 (1.30, 1.81) ^d^
Black non-Hispanic	1.00	1,111	1.00	580	1.00
***Marital status***					
single	1.25 (1.14, 1.38) ^d^	5,780	1.17 (1.05, 1.29) ^c^	5,111	1.21 (1.13, 1.30) ^d^
widowed/divorced/separated	1.19 (1.09, 1.30) ^d^	4,864	1.24 (1.14, 1.35) ^d^	4,791	1.21 (1.14, 1.29) ^d^
unknown	1.21 (0.84, 1.74)	154	1.38 (0.89, 2.11)	107	1.30 (0.99, 1.72)
married	1.00	5,611	1.00	5,733	1.00
***Education (years)***					
9–12	1.24 (0.93, 1.66)	872	1.32 (0.97, 1.79)	832	1.26 (1.02, 1.55) ^b^
13+	1.64 (1.28, 2.11) ^d^	12,816	1.88 (1.45, 2.44) ^d^	12,400	1.72 (1.44, 2.07) ^d^
unknown	1.56 (1.17, 2.08) ^c^	2,180	1.98 (1.47, 2.66) ^d^	1,897	1.75 (1.42, 2.15) ^d^
0–8	1.00	541	1.00	613	1.00
***Military veteran***					
yes	1.09 (0.98, 1.20)	2,789	1.12 (1.02, 1.24) ^b^	13,620	1.11 (1.03, 1.19) ^c^
no/unknown	1.00	3,006	1.00	12,736	1.00
***Region of death***					
other	1.16 (0.92, 1.47)	10,315	1.29 (1.00, 1.66) ^b^	8,675	1.26 (1.01, 1.59) ^b^
South	1.00	6,094	1.00	7,067	1.00
***County urbanicity***					
metropolitan large	1.47 (1.06, 2.03) ^b^	7,830	1.16 (0.83, 1.63)	5,964	1.35 (1.06, 1.71) ^b^
metropolitan small	1.51 (1.11, 2.07) ^c^	5,674	1.21 (0.88, 1.66)	5,810	1.38 (1.10, 1.73) ^c^
metropolitan adjacent	1.22 (0.89, 1.68)	2,350	1.08 (0.79, 1.48)	3,261	1.15 (0.92, 1.45)
micropolitan or adjacent	0.83 (0.52, 1.31)	242	1.08 (0.71, 1.64)	339	0.98 (0.72, 1.34)
rural	1.00	313	1.00	368	1.00
***County poverty (%)***	0.99 (0.98, 1.00) ^b^	16,409	0.96 (0.95, 0.98) ^d^	15,742	0.98 (0.97, 0.99) ^d^

^a^For economy, the table excludes four variables that were incorporated in the multivariable model, but showed no association with the outcome variable. They were chief medical examiner/coroner selection, prior suicide attempt, recent crisis, and emergency medical services at death scene.

^b^ p < 0.05

^c^ p < 0.01

^d^ p < 0.001

## Discussion

Our findings caution users against uncritically taking suicide and other official data at face value. Under-ascertainment means suicide undercounting. Indicative of differential undercounting by method, increased odds of a suicide note manifested both for cases of suicides by drug intoxication and by other poisoning relative to their referent, firearm cases, as consonant with the first hypothesis concerning the relative violence and forensic overtness of methods. Hanging suicides did not deviate from the referent, nor did the combined category of jumping and drowning suicides. The observed note excess for both drug-intoxication and other poisoning suicides persisted through application of each of the multivariable models, including the full multilevel, multivariable model that incorporated autopsy status.

The study implication of growing suicide misclassification and suicide undercounting among drug intoxication deaths assumes added importance as an impediment to prevention in the face of the burgeoning opioid mortality epidemic [50] and severely under-resourced and overburdened emergency healthcare [51,52] and death investigation systems [53,54]. Although plausibly a gross underestimate, at 1.62 per 100,000 population, the drug intoxication suicide rate was 41% higher in 2015 than in 2000 [10]. However, suicides involving such poisons as gases, vapors, metals, pesticides, and household cleaners have remained relatively less common. At 0.50 per 100,000, the rate of non-drug poisoning suicides in 2015 was 12% lower than the rate in 2000. Moreover, determination of intent in the non-drug poisoning cases often rests on the unmistakable actions required to use these suicide methods (e.g., carbon monoxide poisoning where the decedent was found in a car with a hose from the tailpipe to the passenger compartment, windows sealed with duct tape, ignition key in the on position, and the gas tank empty), which diminishes the potential ambiguity they pose for medical examiners and coroners. On the other hand, the literature provides no evidence drug intoxication suicides are more planned and less impulsive than suicides by more overtly violent methods.

As hypothesized, performance of a forensic autopsy attenuated the observed associations between overtness of suicide method and odds of an evidentiary suicide note. Although an autopsy can help investigators identify the mechanism or cause of an injury death, it does not invariably enlighten decedent intentionality [55]. Indeed, performance of an autopsy diminished but did eliminate the note-effect we found when comparing mechanisms of deaths in the manner of death determination. Presence of a suicide note, similar to the effect of an overt suicide method, may even reduce autopsy occurrence [56]. During 2007, 97% of US homicides were autopsied compared with 60% of suicides, 81% of undetermined intent deaths, and 79% of unintentional (accidental) poisoning deaths, pointing to a less rigorous approach to suicide versus homicide determination and accounting [57].

The US *National Association of Medical Examiners* (NAME) *Forensic Autopsy Performance Standards* require an autopsy when “the death is by apparent intoxication by alcohol, drugs, or poison, unless a significant interval has passed, and the medical findings and absence of trauma are well documented” [58]. NAME does not require autopsy in other suicidal deaths where the cause is externally manifest (e.g., gunshot wound or hanging) and instead leaves autopsy performance to the discretion of the pathologist, if it is “necessary to determine cause or manner of death, or document injuries/disease, or collect evidence.” While this standard is expected for medical examiners, who work in some states, it does not apply to elected coroners–the norm for many states. Other jurisdictions have both coroners and medical examiners, varying by counties [39].

By comparison with the US, Finnish medicolegal authorities conducted forensic autopsies in 99% of suicides, 98% of homicides, 98% of unintentional poisoning deaths, and 97% of undetermined deaths in the period 2000–2003 [59]. Researchers viewed a lower autopsy rate and greater use of ill-defined and unknown cause-of-death codes as especially problematic for the quality of Danish suicide and other manner-of-death statistics relative to those of Finland [60,61]. During the period 1998–2007, Finland and Denmark had annualized suicide rates of 20 and 12 per 100,000 population, respectively [62]. Corresponding combined clinical and forensic autopsy rates for total deaths were 31% and 8%. This gap—as a plausible indicator of different standards of thoroughness in medicolegal death investigations [63]—may mean there is an artifactual component in the differential in suicide rates between these two Scandinavian countries. However, substantive factors contributing to a higher rate in Finland might include variable alcohol consumption and firearm ownership between Finland and Denmark.

More generally implicating an artifactual component in suicide rates and rate variation, an ecological or correlational study of 35 Eurasian countries reported spatial and temporal associations between the respective magnitudes of the combined clinical and forensic autopsy rate and the suicide rate [62]. Spatially or cross-sectionally, a 1% difference in autopsy rates was associated with a suicide rate difference of 0.49 per 100,000 population, and temporally or longitudinally a 1% decrease in the autopsy rate with a suicide rate decrease of 0.42 per 100,000.

Our research, examining registered or known suicides, complements another multilevel (individual/county), multivariable study which used NVDRS data from the same observation period to predict differential odds that suicides pooled with deaths of undetermined intent, included as possible suicides, would be classified by medical examiners and coroners as suicide if there was documentation of a suicide note and mental health antecedents [20]. One hypothesis in the complementary study addressed the association between an evidentiary suicide note and suicide classification. Underscoring a pivotal role an authenticated note can play in separating suicide from undetermined cases, presence was associated with 34-fold increased odds of a suicide classification. In addition, combined firearm and hanging/suffocation deaths showed 42-fold increased odds of a suicide classification relative to drug intoxication deaths or, alternatively expressed, 98% lower odds of an undetermined classification. Drug intoxication cases with an evidentiary note were 45 times more likely to be classified as suicide compared to corresponding cases with no note or unknown note status, and eight times more likely in firearm and hanging cases. A relative strength of the current multilevel study, since it focused on suicides rather than suicides and undetermined deaths, was greater granularity and specificity of suicide methods or injury causes/mechanisms than in the comparative NVDRS study.

Based on medicolegal and police investigations, which often involve family and friends of the decedent, NVDRS data are vulnerable to reporter bias, especially among persons who are ashamed or embarrassed by what transpired. This deficiency also may reflect a lack of willingness of investigators to collect data about decedent background and circumstances when suicide is readily apparent as the manner of death, or when as an elected local official, coroners are reluctant to probe potentially sensitive personal issues. Our study and the complementary NVDRS investigation jointly reveal a need for qualitative as well as quantitative research on whether family and friends of the decedents variably destroy or otherwise conceal suicide notes, and withhold other potential corroborative evidence from authorities. The field now would benefit from mixed methods investigations that integrate psychological autopsies, focus groups, surveys, sociocultural autopsies, content analysis, and thematic analysis to examine values and attitudes towards suicide that may continue to influence its reporting. Although high quality data regarding suicides are essential for planning, implementing, and evaluating suicide prevention programs, few resources have been devoted to improving fundamental data quality. Without such rigor, it will be difficult to accept the validity of future efforts to reduce suicide rates.

The enriched Restricted Access Database of the NVDRS provides the only population-based data in the US appropriate for evaluating our research questions about the relationship between suicide methods, a forensic autopsy, and an evidentiary suicide note. Besides reporter bias, a limitation of this study was restriction of the geographic domain to the 17 states that contributed data to the NVDRS throughout the observation period, 2011–2013. Nevertheless, high demographic concordance between these states and the nation [20] tempers our concern about reduced generalizability of study findings, which additionally is a by-product of system protocols that emphasize uniform definitions of manner of death and consistent data collection, entry, review, and coding [44]. Moreover, the similarity in the distribution of suicides by method across age, sex, and race/ethnicity, between the NVDRS states and the nation, enhanced our confidence in study generalizability. Since our observation period, the NVDRS has expanded to 40 states, the District of Columbia, and Puerto Rico, with a goal to cover all 50 states and the territories [64]. Further study limitations included the indirect nature of our assessment of differential suicide data quality by method, confinement of our study population to suicides whose death circumstances were captured by the NVDRS and whose methods were specified, and our inability to factor in medicolegal use of a computerized tomography scan in lieu of a forensic autopsy. Other limitations and strengths of the NVDRS have been reported [20,44,65–67].

## Conclusions

Suicide requires substantial affirmative evidence to establish manner of death, and affirmation of drug intoxication suicides appears to demand an especially high burden of proof. Findings and their implications argue for more stringent investigative standards, better training, and more resources to support accurate and comprehensive case ascertainment, as the foundation for developing evidence-based suicide prevention initiatives.
